# Nickel‐Substituted Rubredoxin Undergoes Demetallation Upon Reaction With Nitric Oxide and Nitroxyl

**DOI:** 10.1002/cbic.70548

**Published:** 2026-09-29

**Authors:** Eduardo H. S. Sousa, Thiago Maciel Bastos, Ana C. S. Gondim, Wellinson G. Guimarães, Marcelo H. M. da Silva, Marta S. P. Carepo, Sofia R. Pauleta, Isabel Moura, José J. G. Moura

**Affiliations:** ^1^ Bioinorganic Group Department of Organic and Inorganic Chemistry Federal University of Ceará Fortaleza Brazil; ^2^ LAQV REQUIMTE Departamento de Química Faculdade de Ciências e Tecnologia Universidade Nova de Lisboa Caparica Portugal; ^3^ Escola de Psicologia e Ciências da Vida Departamento de Ciências da Vida Universidade Lusófona Lisboa Portugal; ^4^ Microbial Stress Lab UCIBIO Departamento de Química Faculdade de Ciências e Tecnologia Universidade Nova de Lisboa Caparica Portugal; ^5^ i4HB Departamento de Química Faculdade de Ciências e Tecnologia Universidade Nova de Lisboa Caparica Portugal

**Keywords:** nickel‐substituted center, nitric oxide, nitroxyl, reactivity, rubredoxin, spectroscopy

## Abstract

Nitric oxide (NO) and nitroxyl (HNO) are closely related reactive nitrogen species with distinct biological functions, yet differentiating their chemistry remains challenging because of their high reactivity and overlapping reaction pathways. Developing simple protein‐based platforms to study their reactivity is therefore of considerable interest. Rubredoxins are small, stable iron–thiolate proteins widely used as model systems for investigating metal–thiolate coordination and electron transfer. Here, we investigated the reactivity of native iron‐rubredoxin (Fe‐Rd) and its nickel‐substituted analog (Ni‐Rd) toward NO and HNO to determine whether cysteine ligand modification could disrupt metal–thiolate coordination and induce demetallation. Electronic absorption and fluorescence spectroscopy showed that Fe‐Rd is highly resistant to both species, whereas Ni‐Rd exhibited pronounced spectral changes consistent with metal release, including loss of the Ni─S ligand‐to‐metal charge‐transfer band and increased intrinsic fluorescence. Inductively coupled plasma atomic emission spectroscopy confirmed partial nickel release following treatment with both NO and HNO donors. Despite demetallation, circular dichroism, thermal denaturation, and size‐exclusion chromatography demonstrated that Ni‐Rd largely retains its secondary structure, thermal stability, and monomeric state. These findings establish Ni‐Rd as a robust minimal metalloprotein platform for probing reactive nitrogen species‐induced thiolate chemistry.

## Graphical abstract

Rubredoxin is a thermally stable thiolate iron‐bound protein that can be prepared as a nickel‐substituted one. This nickel protein showed sensitivity to reaction with NO and HNO donors, causing its demetallation. Notably, these reactions exhibit spectroscopic profiles in the ultraviolet–visible and fluorescence with potential bioanalytical use.

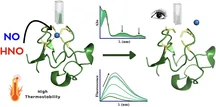

**Abbreviations**
AS: Angelis’ salt (HNO donor)
DEANONOate: diethylammonium (Z)‐1‐(*N*,*N*‐diethylamino)diazen‐1‐ium‐1,2‐diolate (NO donor)
Fe‐Rd: iron‐bound rubredoxin
Ni‐Rd: nickel‐bound rubredoxin

## 1 Introduction

Nitric oxide (NO) and nitroxyl (HNO), the one‐electron reduced and protonated congener of NO, are reactive nitrogen species that play important yet some distinct roles in biological signaling, oxidative stress responses, and redox regulation [1, 2]. Although they are closely related chemically, they exhibit markedly different reactivities toward biological targets, particularly cysteine residues. NO primarily promotes reversible *S*‐nitrosation through nitrosothiol formation, whereas HNO reacts more rapidly with thiolates to produce distinct sulfur‐containing products, including sulfinamides [3, 4, 5, 6]. These differences underlie their diverse biological functions but also make their selective detection challenging because both species are highly reactive, short‐lived, and readily transformed under physiological conditions. Consequently, there is considerable interest in developing simple molecular platforms that can probe their chemistry and provide mechanistic insight into their interactions with sulfur‐containing biomolecules.

Metal–thiolate centers are particularly attractive systems for studying reactive nitrogen species because their reactivity is governed by the interplay between the metal ion and coordinated cysteine ligands. In many metalloproteins, modification of cysteine residues can alter metal coordination and redox properties or even induce metal release, thereby affecting protein function. However, the structural complexity of most metalloproteins often complicates the mechanistic analysis. Minimal metalloproteins with well‐defined metal–thiolate coordination environments therefore offer valuable opportunities to isolate and investigate the fundamental chemistry of reactive nitrogen species.

Rubredoxins are among the simplest naturally occurring metal–thiolate proteins. These small (≈6 kDa) nonheme iron proteins contain a single metal ion tetrahedrally coordinated by four cysteine residues (Cys_4_), forming one of the best‐characterized biological metal–thiolate centers [7, 8]. Their compact architecture, exceptional thermal and chemical stability, and the absence of additional cofactors have established rubredoxins as benchmark systems for investigating metal–thiolate bonding, electron transfer, and metalloprotein structure–function relationships. The rubredoxin from *Desulfovibrio gigas* is particularly well characterized, with detailed structural, spectroscopic, and biochemical information available [7, 8, 9, 10].

Beyond serving as model electron‐transfer proteins, rubredoxins have become versatile scaffolds for metalloprotein engineering. Replacement of the native iron with alternative transition metals, including nickel, cobalt, copper, zinc, and molybdenum, has enabled systematic investigations of metal‐dependent electronic structure, spectroscopy, and reactivity [11, 12, 13, 14, 15, 16]. In particular, nickel‐substituted rubredoxin has emerged as an attractive minimal model for nickel hydrogenases, reproducing key aspects of hydrogen activation and serving as the basis for artificial metalloenzymes and photocatalytic systems [17, 18, 19, 20, 21]. These studies demonstrate that the rubredoxin scaffold tolerates extensive metal substitution while preserving its remarkable structural integrity, making it a robust platform for introducing new chemical functions.

Rubredoxin‐like domains are also found in proteins involved in oxidative and nitrosative stress responses. For example, flavodiiron proteins contain a C‐terminal rubredoxin‐like domain that participates in electron transfer during the detoxification of oxygen and nitric oxide [22, 23, 24]. Moreover, rubredoxin‐derived iron nitrosyl complexes have recently been developed as photoactivatable NO donors [25], and rubredoxin has been implicated in electron‐transfer pathways associated with nitric oxide metabolism [26]. These observations suggest that the rubredoxin scaffold is intrinsically relevant to reactive nitrogen species chemistry. Nevertheless, despite its widespread use as a model metalloprotein and its demonstrated versatility in protein engineering, the direct reactivity of native and metal‐substituted rubredoxins toward NO and HNO has not been systematically investigated.

Here, we address this knowledge gap by examining the reactions of native iron‐rubredoxin (Fe‐Rd) and nickel‐substituted rubredoxin (Ni‐Rd) with NO and HNO donors. We hypothesized that modification of the coordinating cysteine thiolates by reactive nitrogen species would destabilize the metal–thiolate coordination sphere, promoting demetallation while preserving the highly stable protein scaffold. Using complementary electronic absorption, fluorescence, circular dichroism, size‐exclusion chromatography, and ICP‐AES measurements, we investigated both the spectroscopic signatures and structural consequences of these reactions. We show that, whereas Fe‐Rd is remarkably resistant to both reactive nitrogen species, Ni‐Rd undergoes partial demetallation accompanied by pronounced optical responses without significant disruption of its overall fold. These findings establish nickel‐substituted rubredoxin as a minimal and robust metalloprotein platform for investigating reactive nitrogen species‐induced metal–thiolate chemistry and provide a foundation for the development of protein‐based spectroscopic probes for NO and HNO.

## 2 Materials and Methods

### 2.1 Materials

All experiments were performed at 4 °C and pH 7.6, unless otherwise stated. Buffers and reagents were of the highest commercially available grade and prepared in ultrapure water (Milli‐Q). FeCl_2_·4H_2_O was purchased from Merck. Luria–Bertani (LB) medium, ampicillin, isopropyl‐β‐D‐thiogalactopyranoside (IPTG), Tris base, Bradford reagent (Bio‐Rad), Tricine, glycerol, APS, TEMED, agarose, Coomassie Blue, NiCl_2_·6H_2_O, PMSF, reduced glutathione, EDTA, DNase, ethanol, and all other chemicals were purchased from Fisher Scientific and Sigma‐Aldrich and used as received, unless otherwise indicated. Angeli’s salt and DEANONOate stock solutions were prepared by dissolving each solid compound in 10 mM NaOH solution. The concentrations of the stock solutions at 16 mM of each donor were determined by UV–visible absorption spectroscopy using their corresponding molar extinction coefficients of 8000 mol^−1^ L cm^−1^ at 250 nm for Angeli’s salt [27] and 6500 mol^−1^ L cm^−1^ at 250 nm for DEANONOate [28] and further diluted in 1 mM NaOH for use. The actual concentration of the donor stock was measured before and also after each experiment while the stocks were kept on ice during measurements. The metal complex *cis*‐[Ru(bpy)_2_(SO_3_)NO](PF_6_) was prepared and characterized by NMR, FTIR, and cyclic voltammetry as previously reported and used in the experiments described below [29, 30].

### 2.2 Protein Expression, Purification, Demetallation, and Metal Reconstitution

Fe‐ and Ni‐containing rubredoxin (Fe‐Rd and Ni‐Rd) from *D. gigas* were expressed heterologously in *E. coli* BL21(DE3) (Merck) cells transformed with pSPRd containing the rubredoxin gene [31]. Fe‐Rd was produced and purified according to the literature [31], while Ni‐Rd expression followed another reported protocol [18]. Protein purity was assessed by SDS‐PAGE and electronic absorption spectroscopy. Holo Fe‐Rd showed an *A*_280nm_/*A*_495nm_ ratio of 2.3, and Ni‐Rd displayed an *A*_275nm_/*A*_448nm_ ratio of 4.7. Proteins were concentrated, aliquoted, and stored at −80 °C until further use.

### 2.3 Electronic Absorption Spectroscopy

Absorption spectra were recorded in a 1‐cm quartz cuvette using an HP 8453 diode array spectrophotometer with a thermoregulated water bath. Spectra were collected over 190–1000 nm at 26 or 37 °C. Kinetic measurements were performed for up to 1 h, with baseline corrections applied as necessary.

### 2.4 Fluorescence Spectroscopy

Fluorescence was measured using a QM‐40 spectrofluorimeter (PTI) in a 1‐cm quartz cuvette. Excitation was set at 280 nm (10 nm slit width), and emission was monitored from 290 to 420 nm. Measurements were conducted in 50 mM sodium phosphate buffer, pH 7.0, containing 50 mM NaCl, unless otherwise specified.

### 2.5 Electronic Spectroscopy Studies With NO/HNO Donors

Measurements of the reactivity and apparent rate of reaction of Fe‐Rd and Ni‐Rd at 20–25 µM with NO (DEANONOate, diethylammonium (Z)‐1‐(*N*,*N*‐diethylamino)diazen‐1‐ium‐1,2‐diolate) and HNO donors (Angeli’s salt, sodium α‐oxohypo‐nitrite) at substoichiometric (0.5 molar ratio) and overstoichiometric (4 molar ratio) amounts were followed by UV–visible spectroscopy in 50 mM phosphate buffer, pH 7.0, containing 50 mM NaCl, at 26 °C for 80 min. Reactions were also investigated at 37 °C. Potential interferents of the reaction with Ni‐Rd were investigated by incubating the protein with 1 mM sodium nitrite, 1 mM sodium nitrate, 1 mM EDTA, or 2 mM reduced glutathione for 1 h at 37 °C. Titration of 25 µM Ni‐Rd was carried out with NO or HNO donors in 50 mM sodium phosphate buffer, pH 7.0, containing 50 mM NaCl, by sequential addition of known amounts of up to 50 µM at 37 °C.

### 2.6 Fluorescence Measurements

Protein (ca. 5 µM) was prepared in 50 mM phosphate buffer, pH 7.0, with 50 mM NaCl. NO or HNO donor concentrations were added at 5.0, 7.5, 10.0, 12.5, and 15.0 µM. Samples were excited at 280 nm (1 mm slit), and emission spectra were collected from 290 to 420 nm at 26 °C. Donors were added sequentially and incubated ca. 10 min between readings, and the total donor volume remained <5% of the sample volume.

### 2.7 Analytical Gel Filtration Chromatography

Gel filtration was performed on a Shimadzu UHPLC using a TSKgel QC‐PAK GFC 300 column (15 cm × 7.8 mm, 250 Å; Tosoh). Standard proteins were first analyzed to construct a calibration curve of retention volume versus log molecular weight (gel filtration standard, Bio‐Rad). Rd‐Fe and Ni‐Rd (2.5–80 µM) were analyzed alone and with stoichiometric excess of NO or HNO donors incubated at 37 °C, injected after at least 20 min of incubation. The column was equilibrated with 50 mM sodium phosphate buffer containing 50 mM NaCl, pH 7.0, filtered through a 0.45‐µm nylon membrane.

### 2.8 Circular Dichroism (CD)

Far‐UV CD spectra were recorded to assess the secondary structure using a J‐815 spectropolarimeter (Jasco, Japan), with Ni‐Rd samples at 8 µM (far‐UV) and 60 µM (near‐UV). Samples were prepared in 5 mM sodium phosphate buffer, pH 7.0. Donors were added in 2‐fold molar excess and were incubated for 60 min at 37 °C before spectra were recorded (accumulation of 10 scans). Thermal stability was measured by running assays with 8 µM or 60 µM Ni‐Rd and postreaction of 8 µM or 60 µM Ni‐Rd treated with a 2‐fold molar excess of NO or HNO, following changes at 200 nm from 10 to 90 °C with a heating rate of 1 °C min^−^^1^.

### 2.9 ICP‐AES Measurements

Metal content was determined in the Laboratório de Análises (LAQV) using a Horiba Jobin‐Yvon Ultima ICP‐AES (France) equipped with a 40.68 MHz RF generator, 1 m Czerny–Turner monochromator, AS500 autosampler, and CMA. Samples were prepared in CHELEX‐treated 20 mM HEPES buffer. Ni‐Rd (40 µM) was reacted with a 10‐fold excess of DEANONOate or Angeli’s salt at 37 °C for 4 h. After the reaction, samples were concentrated over a 3‐kDa molecular weight cutoff membrane Centricon, and the concentrated and flow‐through collected were analyzed. Ni quantification was performed using calibration standards (0.31–5 mg L^−1^). Further details can be found in the Supporting Information.

### 2.10 Assay With Metal–NO/HNO Donor [Ru(bpy)_2_(SO_3_)(NO)](PF_6_)

Reactions were conducted with 25 µM Ni‐Rd, 25 µM[Ru(bpy)_2_(SO_3_)(NO)](PF_6_) (from a 2 mM stock of this compound dissolved in water), and 2 mM glutathione in 50 mM phosphate buffer pH 7.0 with 50 mM NaCl. This reaction was monitored for 40 min at 37 °C. In a separate assay, Ni‐Rd with [Ru(bpy)_2_(SO_3_)(NO)](PF_6_) at the same molar ratios was irradiated with UV light (*λ*_max_ = 395 nm, 50 W) for 60 min at 26 °C, recording spectra every 5 min.

## 3 Results and Discussion

### 3.1 HNO and NO Effects on Transition Electronic Bands of Rubredoxin

*D. gigas* rubredoxin was heterologously produced with a yield of ca. 2 mg/L of bacterial culture. The purified protein exhibited the feature of the native and previously isolated using the same procedure, containing 0.9:1 Fe/protein (ca. 91% Fe and 9% Zn^2+^). This protein can be harshly treated to remove native iron as described in the literature [15], followed by reconstitution with other metal ions (e.g., Ni^2+^) [15]. Here, we employed an alternative strategy, developed by Naughton et al. [18], where a nickel‐reconstituted rubredoxin was prepared during heterologous production in *E. coli*. This protein sample showed reliability (yield of 5 mg L^−^^1^ of culture), and its Ni content was over 95%, as indicated by UV–vis and ICP‐AES (Figure S1). The electronic spectra of the iron‐rubredoxin and nickel‐rubredoxin were fully consistent with previous studies [15] and also in agreement with Naughton et al.’s studies [18].

In this work, we employed two well‐known donors of NO and HNO, DEANONOate and Angeli’s salt, respectively, to investigate their effects on iron‐ and nickel‐rubredoxins. These donor compounds are rapid releasers of NO (*t*_1/2_ = 2 min at pH 7.4 and 37 °C [32]) and HNO (*t*_1/2_ = 2.3 min under the same conditions) [32], enabling the exploration of their impact on metal centers by taking advantage of the high, transient flux of these species. Here, iron‐ and nickel‐rubredoxins were used as a model for metalloproteins, aiming to better understand the reactivity of tetrahedral metal–sulfur‐based sites.

NO is well known for its ability to disassemble iron–sulfur proteins, a process that serves as a mechanism for sensing this molecule in vivo [33, 34]. However, some iron–sulfur proteins are resistant to NO treatment, such as iron‐rubredoxin from *Pyrococcus furiosus* [25]. It is also important to consider the distinct reactivity of multinuclear iron–sulfur clusters compared to mononuclear iron sites [33], such as those present in rubredoxins. As far as we are aware, there is no study investigating the reactivity of rubredoxin with HNO so far, and only one report studied the reactivity of *P. furiosus* iron‐rubredoxin with NO. This evident lack of investigations reinforces the importance of better understanding such reactivities from the fundamental aspect to their potential applications.

Interestingly, the native iron‐containing *D. gigas* rubredoxin (Fe‐Rd) was insensitive to both NO and HNO donors, even at relatively high concentrations (stoichiometric excess) and after prolonged incubation (1 h) at 37 °C, as evidenced by the absence of changes in its electronic absorption bands (Figure S2). This behavior highlights the remarkable robustness of the protein, in which the iron center is tightly coordinated by the thiolate groups of four cysteine residues. Indeed, previous studies on *P. furiosus* rubredoxin have shown that NO binding to rubredoxin requires disruption of this coordination sphere, as a mutant protein lacking one of the four cysteine ligands was necessary to enable NO binding [25]. In addition, we should remark that even in that case, iron was still held in place, allowing NO to be bound directly to iron [25], indicating its strong binding to thiolates in rubredoxin.

In contrast, *D. gigas* nickel‐rubredoxin (Ni‐Rd) exhibited clear spectral changes in the visible region upon treatment with both HNO and NO donors (Figure 1). To determine the timescale of potential reactions between NO or HNO and Ni‐Rd, we monitored a 20 μM protein solution in the presence of two concentrations of the respective donors, at 25 °C (Figure 1). The presence of either NO or HNO (in a 1:4 ratio) led to a major decrease in the ligand‐to‐metal charge transfer (LMCT) bands within less than 30 or 60 min, respectively. These reactions fit to a first‐order kinetic equation with *k*_obs_ of 0.083 ± 0.001 min^−1^(*R* ^2^ = 0.999) and 0.126 ± 0.001 min^−1^(*R* ^2^ = 0.999) for NO at 10 μM and 80 μM, respectively. Similarly, upon treatment with HNO, a first‐order kinetic fitting was achieved with *k*_obs_ of 0.0331 ± 0.0005 min^−1^(*R* ^2^ = 0.999) and 0.0434 ± 0.0009 min^−1^(*R* ^2^ = 0.998) at 10 μM and 80 μM of the donor, respectively. These observations suggested a possible route of demetallation of Ni‐Rd. Aiming to investigate if a linear reaction trend could be achieved, we carried out a direct titration measurement with the donors, preincubated at 37 °C, which showed promising response, enabling expectations for its bioanalytical application (Figure S3).

**FIGURE 1:**
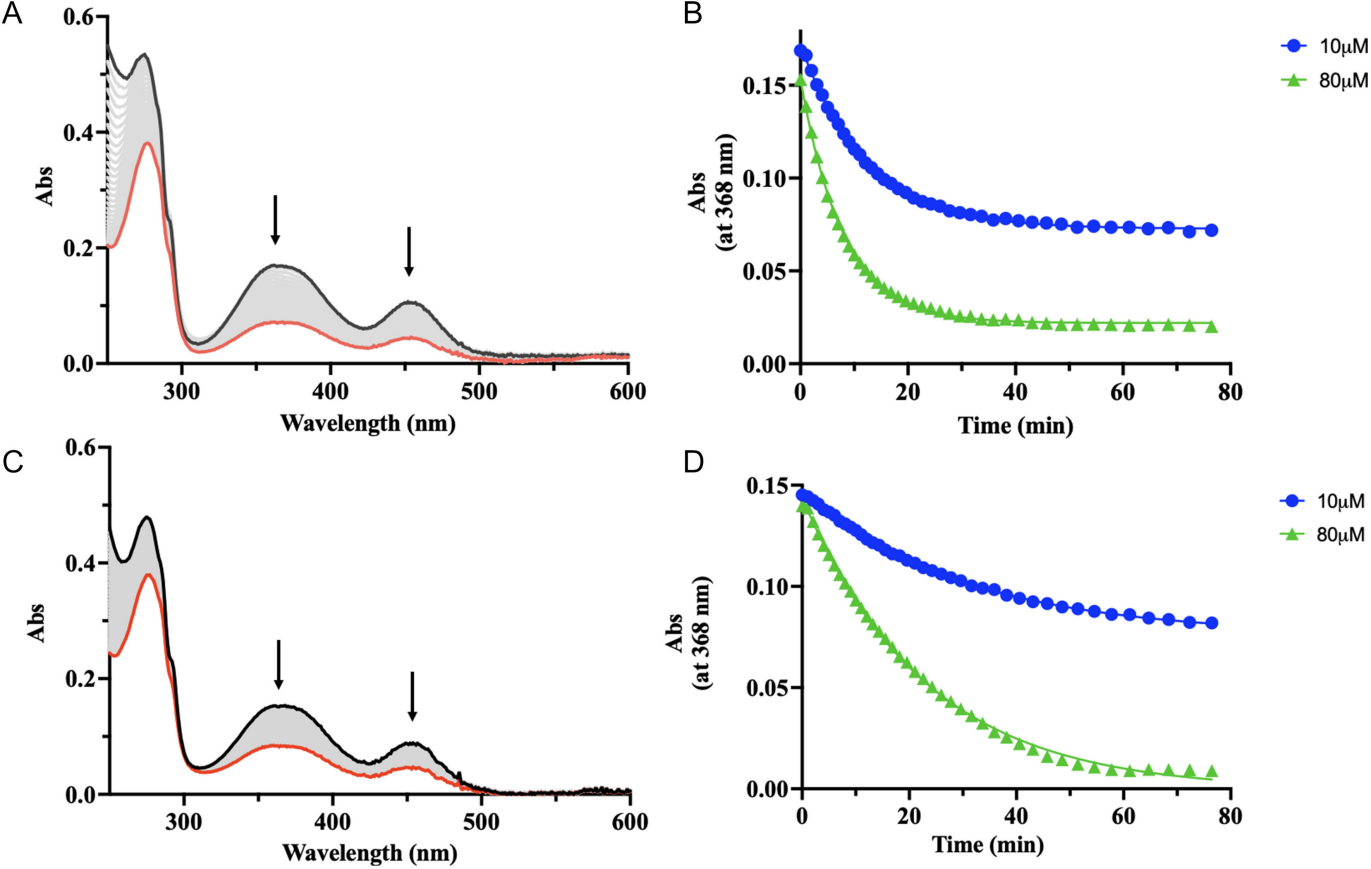
Monitoring the electronic spectrum of Ni‐Rd (20 μM) in 50 mM sodium phosphate buffer pH 7.0, 50 mM NaCl after addition of DEA‐NONOATE (10 μM NO, A) and Angeli’ssalt (10 μM HNO, C) for 80 min. Kinetic plots are represented monitoring changes over time at 368 nm, 25 °C for the reaction with NO (B) and HNO (D) at 10 and 80 μM of donors, which were fit to a first‐order equation (*R* ^2^ ≥ 0.99).

In addition, we investigated some potential interferents that could contribute to or eventually prevent this reaction from occurring. Nitric oxide is known to decompose in an aqueous solution, originating nitrite that can be further oxidized to nitrate. In addition, many studies with NO metallodonors can involve the use of reducing agents, such as glutathione, or the addition of chelators, such as EDTA [30]. Based on that, we investigated if those species could disturb the electronic spectrum of Ni‐Rd, which was monitored for 1 h at 37 °C. Our results showed no significant changes in the electronic spectrum of the protein (Figure S4) reinforcing the lack of reactivity toward those molecules, indicating potential for Ni‐Rd to be further explored in the detection of NO or HNO.

Inorganic chemistry principles can support some of these data, considering that iron(III) shows a “d^5^” electronic configuration system that can better accommodate a high‐spin tetrahedral geometry and engage in a strong covalency with thiolates with ligand‐to‐metal charge transfer than that of nickel(II). The latter shows a “d^8^” electronic configuration exhibiting better ligand field stability in a square planar or even octahedral geometry than tetrahedral. In addition, nickel(II) has a lower charge, also affecting charge transfer and covalency with thiolates. Thus, we would expect that rubredoxin containing iron(III) is indeed less prone to react than rubredoxin with nickel(II). This latter site with a less stable geometry might be more susceptible, in agreement also with other studies including Fe─S and Ni─S bond lengths. Here, we explored the capacity of NO and HNO to react with metal‐bound thiols as a strategy to better understand the stability of metallosites as well.

### 3.2 HNO and NO Effects on the Intrinsic Fluorescence of Nickel‐Rubredoxin

Rubredoxin from *D* . *gigas* contains two phenylalanine (F49 and F30), three tyrosine (Y4, Y11, and Y13), and one tryptophan (W37) residue within a relatively small protein scaffold. The intrinsic fluorescence of these aromatic residues can be efficiently quenched by nearby paramagnetic metal centers (e.g., Fe^3+^, Ni^2+^). Therefore, disruption of the metal site and subsequent metal release are expected to result in an increase in the intrinsic fluorescence of this protein. Besides that, fluorescence changes could also report even minor local conformational changes by exposing those residues to distinct microenvironments, which could result from specific modifications in the cysteine residues (e.g., formation of sulfinamide). The closest residues to the metal center are F49 and Y11 (ca. 5.1–5.3 Å), but the others are still in expressive proximity as well (e.g., W37 at ca. 7.6 Å) being affected by the paramagnetic center (Figure S5). Even though this technique can provide additional indications of demetallation, it could also report further subtle changes. Based on this rationale, we investigated the intrinsic fluorescence of rubredoxin after treatment with NO and HNO donors.

Ni‐Rd exhibits an emission maximum at approximately 318 nm upon excitation at 280 nm. Notably, this signal was significantly affected by the addition of NO and HNO donors (Figure 2). Initially, both donors induced an increase in fluorescence intensity accompanied by a redshift in the emission maximum. This behavior is consistent with the demetallation of rubredoxin, where removal of the paramagnetic quenching center enhances fluorescence, while partial unfolding or structural rearrangements expose aromatic residues to a more polar environment. This overall process may lead to the observed shift to a longer emission wavelength (ca. 332 nm with NO and 326 nm with HNO). This type of behavior has been reported in many other metalloproteins or metal‐binding proteins and also explored for analytical purposes [35, 36, 37, 38].

**FIGURE 2:**
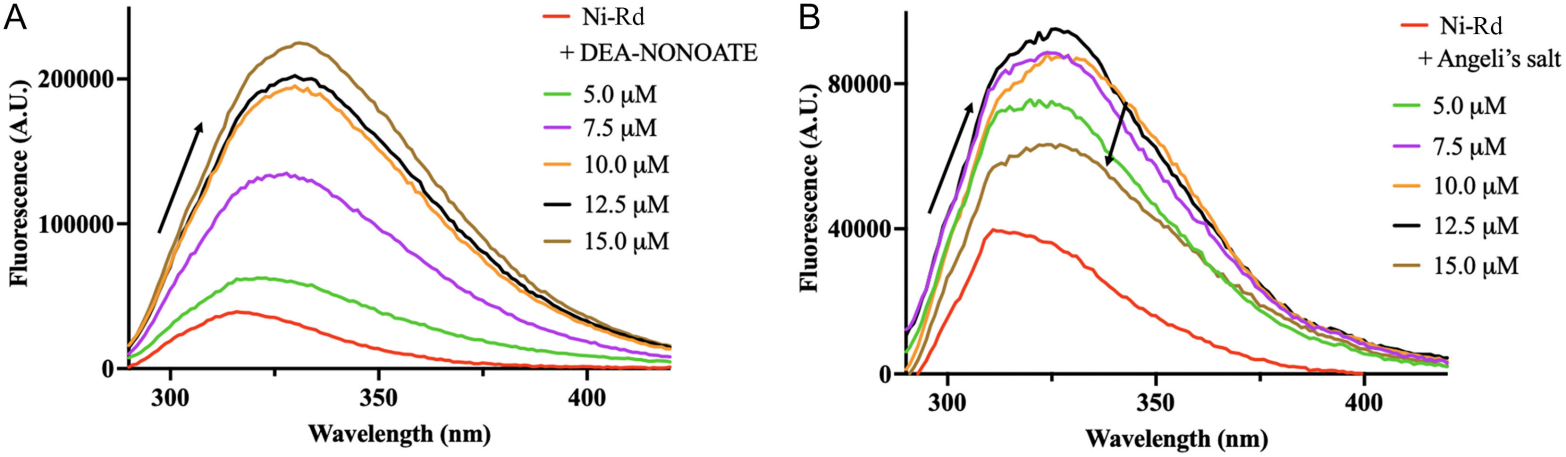
Intrinsic fluorescence of Ni‐Rd at 5 μM in 50 mM sodium phosphate buffer pH 7.0 with 50 mM NaCl, upon treatment with NO (A) or HNO (B) donors; left arrow shows trend until ca. 10 μM, and right arrow from 10 to 15 μM of donor at increasing concentrations. Excitation at 280 nm.

In addition, some differences were observed between the treatment with NO and HNO when an excess of these donors was used. In the presence of NO, even at concentrations exceeding stoichiometric amounts relative to nickel, the fluorescence increased gradually and reached a plateau. This behavior seems consistent with demetallations of Ni‐Rd likely driven by a reaction of NO and HNO with the thiolate ligands. For this reaction, we expect, based on the literature, the formation of nitrosothiols, which are typically unstable intermediates and could even lead to their conversion into disulfide bonds. This process could preserve the modified cysteine site but make it unviable as a metal‐chelating site. Actually, this type of conversion of the nitrosothiol group into disulfide is described when other cysteine residues are nearby or in reaction with small thiol molecules [4]. This type of behavior could potentially generate a minor structural disturbance within the metal‐binding site.

In contrast to the previous profile, HNO induced a progressive increase in fluorescence intensity and a concomitant redshift up to a Ni:HNO ratio at ca. 1:1.2. Beyond this ratio, a decrease in fluorescence intensity was observed, likely reflecting further modification of the cysteine residues due to the expected higher reactivity of HNO toward thiols than NO [1]. Such modifications commonly lead to a reasonably stable sulfinamide functional group that could promote additional local conformational changes that might be less pronounced than in the reaction with NO. Besides that, sulfinamide groups (multiple ones or in combination with nearby cysteine residues) could work as binding motifs, enabling potential interaction with nickel(II). This type of behavior would lead to fluorescence suppression, as noticed by its lower emission intensity at a higher excess of HNO. Unfortunately, we do not have any further data to validate these working hypotheses so far. We should also remark that the maximum fluorescence emission of rubredoxin (ca. 318 nm) showed only a slight redshift upon treatment with HNO (ca. 326 nm) in comparison to NO treatment (ca. 332 nm). This behavior suggests that those modifications may lead to distinct final structural changes, despite promoting an initial analogous event of demetallation as further validated.

Overall, these fluorescence data are in good agreement with the electronic spectroscopy results, where a major decrease of the UV–visible bands agrees with the loss of metal and matches the initial enhancement in the fluorescence signal.

Our observations suggest that the nickel center would make it easier for cysteine modification at higher HNO or NO donor concentrations, leading to distinct fluorescence patterns that are not observed in the native Fe‐Rd protein. These results support the hypothesis that nickel substitution enhances accessibility and sensitivity to reactive nitrogen species, which may be associated with its weaker binding and geometry constraints.

### 3.3 A Nitrosyl Metallocompound as a NO and HNO Donor

In addition to using fast and stoichiometric NO and HNO donors, we also explored the ability of this protein to report the reaction upon the release of such nitrogen‐based species using a nitrosyl ruthenium compound, [Ru(bpy)_2_(SO_3_)NO](PF_6_) [29]. This compound can release NO upon light irradiation [39], while HNO can be mainly produced upon the reaction with glutathione [30]. Light promotes a transient photoreduction of the nitrosyl moiety of this ruthenium compound (MLCT transition band), followed by the release of NO species, as previously investigated [29, 39]. On the other hand, chemical reduction of this metal compound using an excess of glutathione can provide two electrons to the nitrosyl moiety inducing the release of HNO species as detailed elsewhere (Scheme S1), which is also enhanced at 37 °C and might improve the reaction with Ni‐Rd [30]. Some of the authors have been developing such type of compounds and investigating their reactivity [34]. Such versatility for this metallocompound allowed us to explore a single donor system using nickel‐rubredoxin as a probe, which might be an advantage considering that some NO and HNO probes are sensitive to glutathione or not stable with UV‐light irradiation. Indeed, this nitrosyl metal compound showed evidence of promoting a reaction with Ni‐Rd upon stimulation with reduced glutathione and UV light, despite not being as intense as that noticed with the standard donors (Figure 3). Previously, we showed that glutathione is unable to promote changes in Ni‐Rd, while we also investigated if some effect would be caused by direct irradiation of UV light on Ni‐Rd, which was experimentally discarded (Figure S6).

**FIGURE 3:**
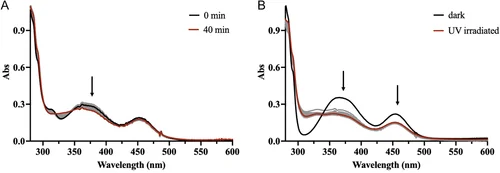
Measurement of the effect of a ruthenium nitrosyl compound ([Ru(bpy)_2_(SO_3_)NO](PF_6_)) as a HNO and NO donor using Ni‐Rd as a probe in 50 mM sodium phosphate buffer pH 7.0 with 50 mM NaCl. (A) Ni‐Rd with [Ru(bpy)_2_(SO_3_)NO](PF_6_) upon addition of 2 mM glutathione to release HNO, monitored for 40 min at 37 °C. (B) Ni‐Rd with [Ru(bpy)_2_(SO_3_)NO](PF_6_) upon UV‐light irradiation to release NO (*λ*_max_ = 395 nm, 60 min) at 26 °C.

In fact, Ni‐Rd can react with HNO and NO, as observed by the decrease in the intensity of the LMCT absorption bands. Despite the effect with glutathione being discreet using the metallodonor, there is a clear reaction trend due to its expected very fast burst release, while light can provide a more continuous release of NO, sustaining that reaction over a longer time. Nevertheless, these measurements supported that Ni‐Rd can still respond to the use of other NO/HNO donor sources and function even under specific distinct stimuli.

### 3.4 Investigating the Structural Effect of NO/HNO Reactivity

The demetallation of Ni‐Rd can be probed by UV–vis spectroscopy as shown before, considering that the loss of nickel(II) disrupts strong LMCT absorption bands (S(pπ)→Ni(II)dπ). However, this structurally localized event may not necessarily affect the overall structure of rubredoxin, which has been reported as a very resilient structure. Interestingly, we should mention that a series of metal ions were used to replace iron(III) in rubredoxin, but these proteins have a similar overall structure [40]. These studies have come to suggest the major role of the protein backbone, which might enforce a defined geometry around all of these metal ions, not the other way around. Based on this, the demetallation process might not necessarily cause major structural changes, but it could still allow minor changes to be noticed with a suitable technique.

Nonetheless, a demetallated rubredoxin with unprotected cysteines is also very reactive and capable of promoting even crosslinks of subunits through intermolecular disulfide bridges, as noticed during nonreducing SDS‐PAGE studies. Notably, modifications of cysteine residues can indeed moderate their reactivities upon the loss of the metal ion. Besides that, we should reinforce that NO and HNO promote distinct modification reactions in the cysteine residues that might result in distinct structural effects, even though both had caused the loss of metal ion.

Aiming to shed further light on the overall structural effect of this reaction, we investigated if any changes occurred disturbing the secondary and tertiary structure of Ni‐Rd upon treatment with NO and HNO donors. Initially, we collected circular dichroism spectra for Ni‐Rd in far‐UV and near‐UV region, where treatment with NO did not seem to cause any significant changes (Figure 4). The absence of detectable CD changes after NO treatment suggests that NO‐induced cysteine modifications do not significantly perturb the global protein fold under our experimental conditions. Indeed, NO under aerobic conditions is known to react with thiols originating transient nitrosothiol groups that, in the vicinity, could decompose into disulfide bridges. This process could lead to a local event with likely minor structural disturbance, explaining our CD results.

**FIGURE 4:**
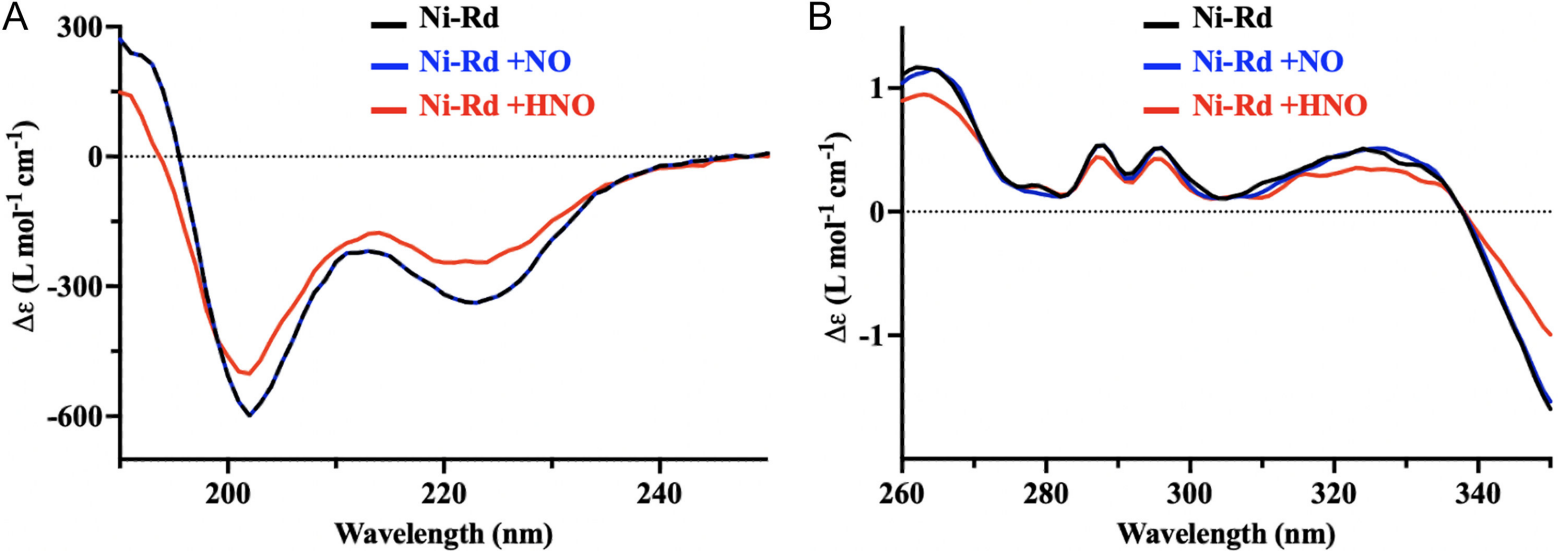
Circular dichroism for Ni‐Rd and treated with NO or HNO (2‐fold molar excess) measured in the far‐UV (A) and near‐UV (B) at 8 and 60 μM of Ni‐Rd in 5 mM sodium phosphate buffer pH 7.0.

Interestingly, treatment of Ni‐Rd with the HNO donor showed some significant changes in both regions of CD spectra (Figure 4). The reaction with HNO, despite leading to a similar demetallation event promoted also by NO, may be motivated by a distinct type of cysteine modification with a much more stable functional group (e.g., sulfinamide). This process could lead to a more significant local structural disturbance, which was reported by CD changes and also in agreement with fluorescence data as well.

In addition, we also looked at differences in thermal stabilities for those samples, where only marginal changes were noticed, particularly upon treatment with HNO. However, nickel‐rubredoxin was still mostly stable even at 95 °C, either with or without treatment with the donors (Figure S7). This study supported that modifications caused by HNO can induce more subtle but noticeable structural effects than with NO. Despite the fluorescence study indicating conformational changes in both cases upon treatment with NO and HNO, their overall behavior suggested that distinct events may take place as expected based on the distinct type of modifications. This process might reflect that demetallation is not a major structural driver, while alteration after that can cause further changes as reported by CD, which could be better noticed with HNO.

In addition to that, we also investigated if NO or HNO could alter the oligomerization state of Rd by running size‐exclusion chromatography (SEC). We should remark that only a loss of the metal ion can leave the cysteine reactive enough to even crosslink with other subunits; then, blockage of these groups could prevent such further events. Indeed, all samples treated or not with the donors showed identical retention times of 5.2 min, supporting that the reaction did not alter its monomeric state or cause any protein degradation (Figure S8). The electronic spectra of the peak of the chromatogram for all samples, including iron rubredoxin, were collected, validating the identity of each species. These results further support that the cysteine residues might be modified.

The spectroscopic data showed evidence for the reaction with NO and HNO, either the disappearance of LMCT bands or redshift‐enhanced fluorescence, but they are not solid evidence for metal removal of the rubredoxin, since a reduction or direct binding to metal (even if unlikely for Ni^2+^) could eventually lead to those changes. Actually, reduction of iron(III) to iron(II) in rubredoxin can lead to major changes in the electronic spectrum, as previously observed [41]. Thus, we carried out a measurement to unveil this process. Here, we employed an ultraconcentrator device (3.5 kDa of cutoff), where we pretreated Ni‐Rd with NO or HNO, then concentrated, and washed the concentrate twice. The protein sample in the concentrator upper vial and the flow‐through in the lower vial were collected, their volumes measured, and their content of nickel checked using ICP‐AES. Notably, in the flow‐through, we found a substantial amount of free Ni^2+^ (ca. 0.2–0.4 ppm or 32%–48% of total), supporting that NO and HNO can indeed remove that metal. Their capacity to remove Ni^2+^ from Ni‐Rd was not complete during this experiment, but NO and HNO donors displaced an average of 32% ± 2% and 48% ± 6% of the metal ion, respectively.

## 4 Conclusions

In this study, we demonstrated that nickel‐rubredoxin is susceptible to reactive nitrogen species, nitric oxide (NO) and nitroxyl (HNO). While the native Fe‐Rd protein showed no response to either species, Ni‐Rd produced clear spectroscopic changes, as reflected in variations in UV–vis absorption and intrinsic fluorescence. Despite the reaction of NO and HNO causing a similar overall effect on the demetallation of Ni‐Rd, a distinct pattern was noticed in fluorescence, indicating likely further modification of the cysteines in the presence of large excess of the donors. This is likely due to the type of cysteine modification promoted by each one of those reactive species, with HNO being responsible for more stable functional groups such as sulfinamide. This study made clearer the robustness of the overall structure of rubredoxin either with Ni(II) or without, but its sensitivity of using thiol‐reactive agents to disrupt its binding site is not achievable with iron(III).

Spectroscopic data indicate that demetallation involves disruption of the metal–thiolate coordination environment, leading to loss of paramagnetic quenching and likely increased exposure of aromatic residues. Nevertheless, the free cysteine residues are either modified or not accessible to form disulfide bonds as Ni‐Rd did not change its oligomeric state.

Overall, these results highlight the potential of small and robust metalloproteins such as rubredoxin as tunable scaffolds for investigating the chemistry of reactive nitrogen species. Ni‐rubredoxins therefore represent promising platforms for the development of simple spectroscopic probes for NO and HNO, with possible applications in biochemical and biotechnological studies.

## Author Contributions

**Eduardo H. S. Sousa** **:** conceptualization, supervision, methodology, investigation, formal analysis, data curation, funding, writing – review and editing. **Thiago** **Maciel** **Bastos:** methodology, investigation, formal analysis, data curation. **Ana C. S.** **Gondim:** methodology, investigation, formal analysis, data curation. **Wellinson** **G.** **Guimarãe** **s** **:** methodology, investigation, formal analysis, data curation, writing – original draft. **Marcelo H. M. da Silva:** methodology, investigation. **Marta S.** **P. Carepo** **:** supervision, methodology, investigation, writing – review and editing. **Sofia R. Pauleta:** supervision, methodology, investigation, writing – review and editing. **Isabel** **Moura:** funding, writing, methodology, investigation, formal analysis, data curation, supervision. **José** **J. G.** **Moura:** funding, writing, methodology, investigation, formal analysis, data curation, supervision.

## Funding

This study was supported by Fundação para a Ciência e a Tecnologia (FCT) (2022.01152.PTDC; UID/04378/2025; UID/PRR/04378/2025; LA/P/0140/2020; UID/50006/2025; UID/PRR/50006/2025 AND LA/P/0008/2020), CAPES (001, 88881.311908/2018‐01, 88881.973965/2024‐01), CNPq (403447/2023‐2, 441090/2023‐0, 441420/2023‐0, 3027756/2022‐1, 308961/2026‐9), FUNCAP (PS1‐0186‐00087.01.00/21), Finep (CV. 01.22.0174.00, CV. 01.23.0033.00).

## Conflicts of Interest

The authors declare no conflicts of interest.

## Supporting information

Electronic spectroscopy measurements of Ni‐Rd and Fe‐Rd, ICP‐AES data and SDS‐PAGE of Ni‐Rd; Monitoring the reaction of Fe‐Rd with NO/HNO by electronic spectroscopy; Titrations of Ni‐Rd with NO and HNO; Monitoring of Ni‐Rd electronic spectra after addition of nitrite, nitrate, EDTA or glutathione; 3D structure of Fe‐Rd highlighting the relative position of fluorescent protein residues;Monitoring of Ni‐Rd electronic spectra upon UV light irradiation; Thermal stability measurements monitored by CD for Ni‐Rd and Ni‐Rd pre‐treated with NO or HNO;Size‐exclusion chromatography measurements for Fe‐Rd and Ni‐Rd, and also with NO and HNO; Scheme 1 for the reactions of release of NO and HNO promoted by [Ru(bpy)2(SO3)NO](PF6); Experimental details of the measurement of Ni(II) by ICP‐AES.
