# Supplementary material for: Nickel‐Substituted Rubredoxin Undergoes Demetallation Upon Reaction With Nitric Oxide and Nitroxyl
Source: Chembiochem. 2026 Sep 29;27(19):e70548. doi: 10.1002/cbic.70548 (PMC13623106; doi:10.1002/cbic.70548)
Supplement: Supplementary file 1 [file CBIC-27-e70548-s001.pdf]

Nickel-Substituted Rubredoxin Undergoes Demetallation upon Reaction with Nitric Oxide and Nitroxyl

Eduardo H. S. Sousa*[1], Thiago Maciel Bastos[1], Ana C. S. Gondim[1], Wellinson G. Guimarães[1], Marcelo H. M. da Silva[1], Marta S. P. Carepo[2,3], Sofia R. Pauleta[4,5], Isabel Moura[2], José J. G. Moura*[2]

1 Bioinorganic Group, Department of Organic and Inorganic Chemistry, Federal University of Ceará, Fortaleza, Brazil

2 LAQV, REQUIMTE, Departamento de Química, Faculdade de Ciências e Tecnologia, Universidade Nova de Lisboa, Caparica, Portugal

[3] Escola de Psicologia e Ciências da Vida, Departamento de Ciências da Vida, Universidade Lusófona, Campo Grande 376, 1749-024 Lisboa, Portugal.

[4] Microbial Stress Lab, UCIBIO, Departamento de Química, Faculdade de Ciências e Tecnologia, Universidade Nova de Lisboa, 2829-516 Caparica, Portugal

[5] i4HB, Departamento de Química, Faculdade de Ciências e Tecnologia, Universidade Nova de Lisboa, 2829-516 Caparica, Portugal

*Corresponding authors: eduardohss@dqoi.ufc.br; jose.moura@fct.unl.pt

Note 1: Protein samples prepared showed metal content measured by ICP-AES as below.

Sample	Ni (mg/L) [relative %]	Zn (mg/L) [relative %]	Fe (mg/L) [relative %]
Ni-Rd	4.25 ± 0.66 [4.6%]	0.206 ± 0.004 [95.4%]	-
Fe-Rd	-	0.054 ± 0.004 [9.2%]	0.534 ± 0.008 [91%]

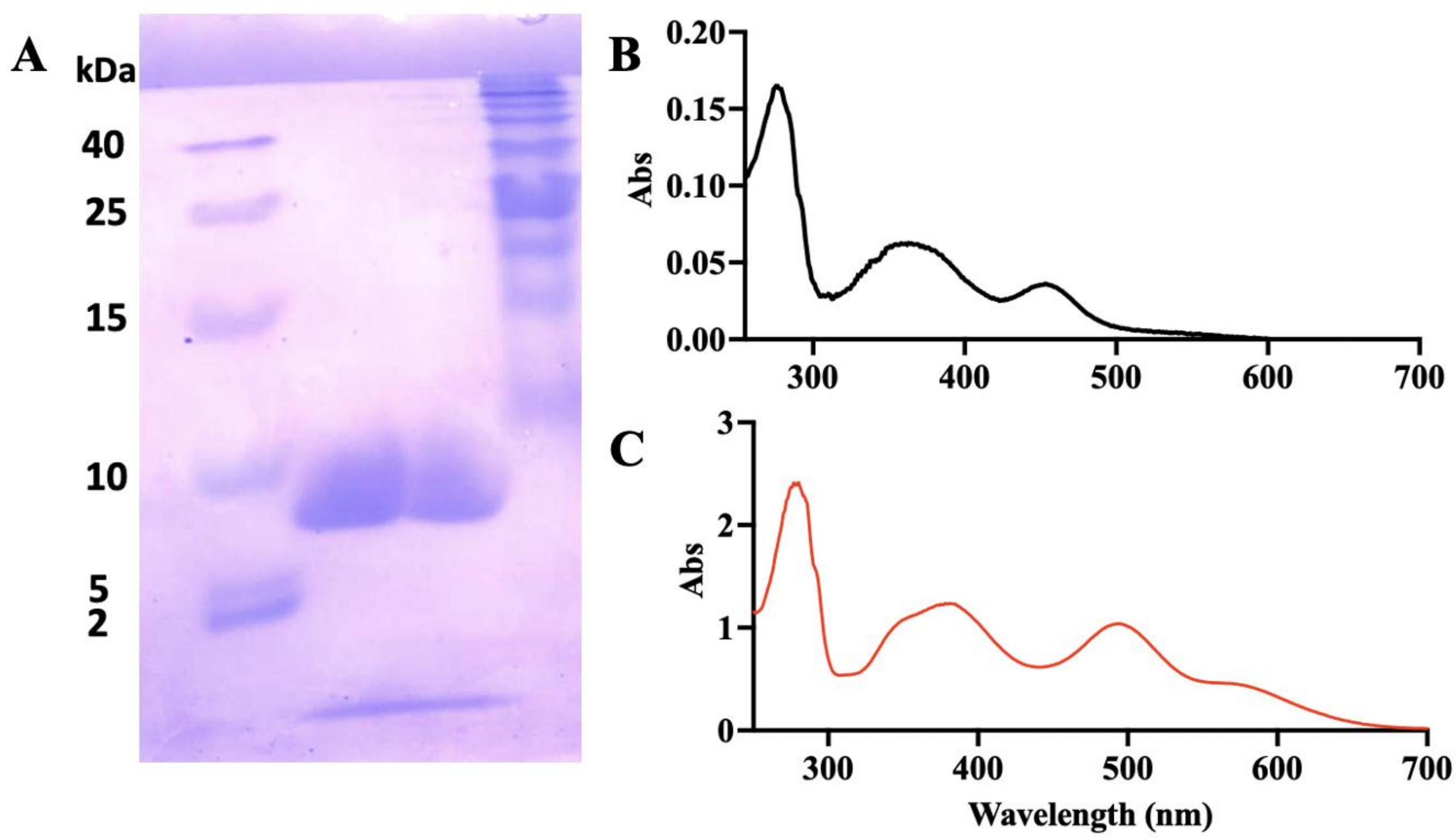

Figure S1. Protein purification and characterization: 17% SDS-PAGE of purified Ni-Rd (A), electronic spectra of purified rubredoxin proteins as Ni-Rd (B) and Fe-Rd (C).

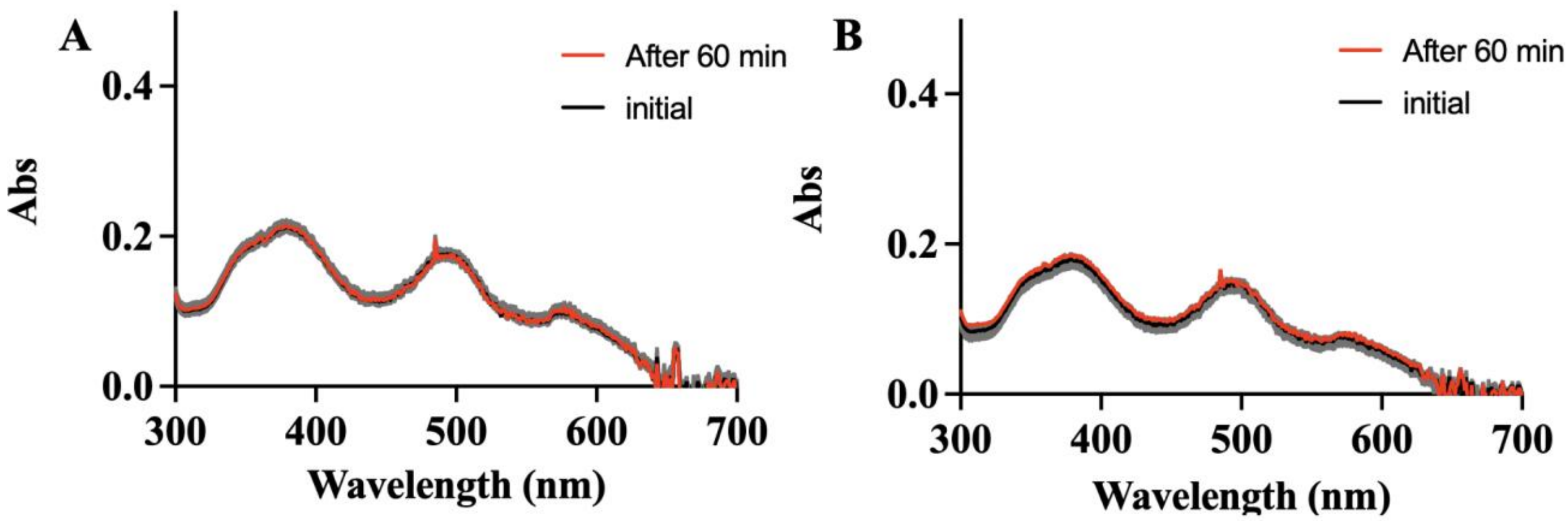

Figure S2. Monitoring the effect of NO and HNO donors at 40 μM with Fe-Fe (20 μM) by electronic spectroscopy, in 50 mM sodium phosphate buffer pH 7.0 with 50 mM NaCl, for 1 h at 37 ^{o}C.

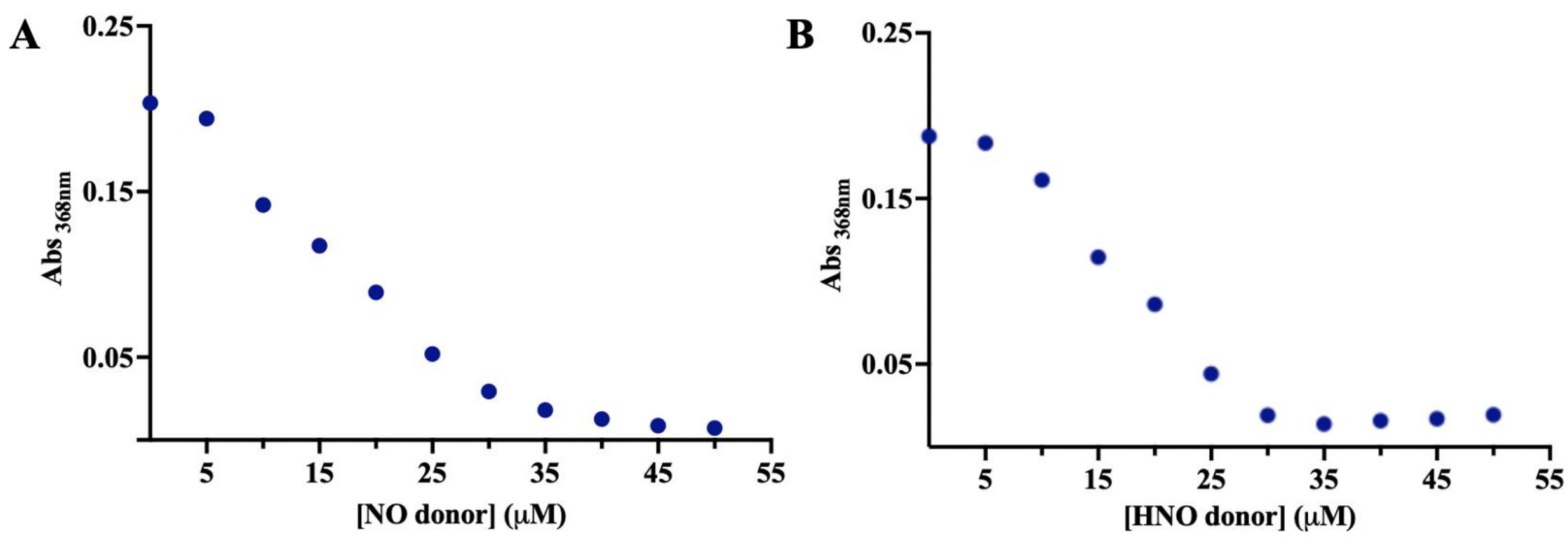

Figure S3. Titration of Ni-Rd (25 μM) with NO (A) and HNO (B) donors monitored by electronic spectroscopy (at 368 nm), in 50 mM sodium phosphate buffer pH 7.0 with 50 mM NaCl, at 37 ^{o}C.

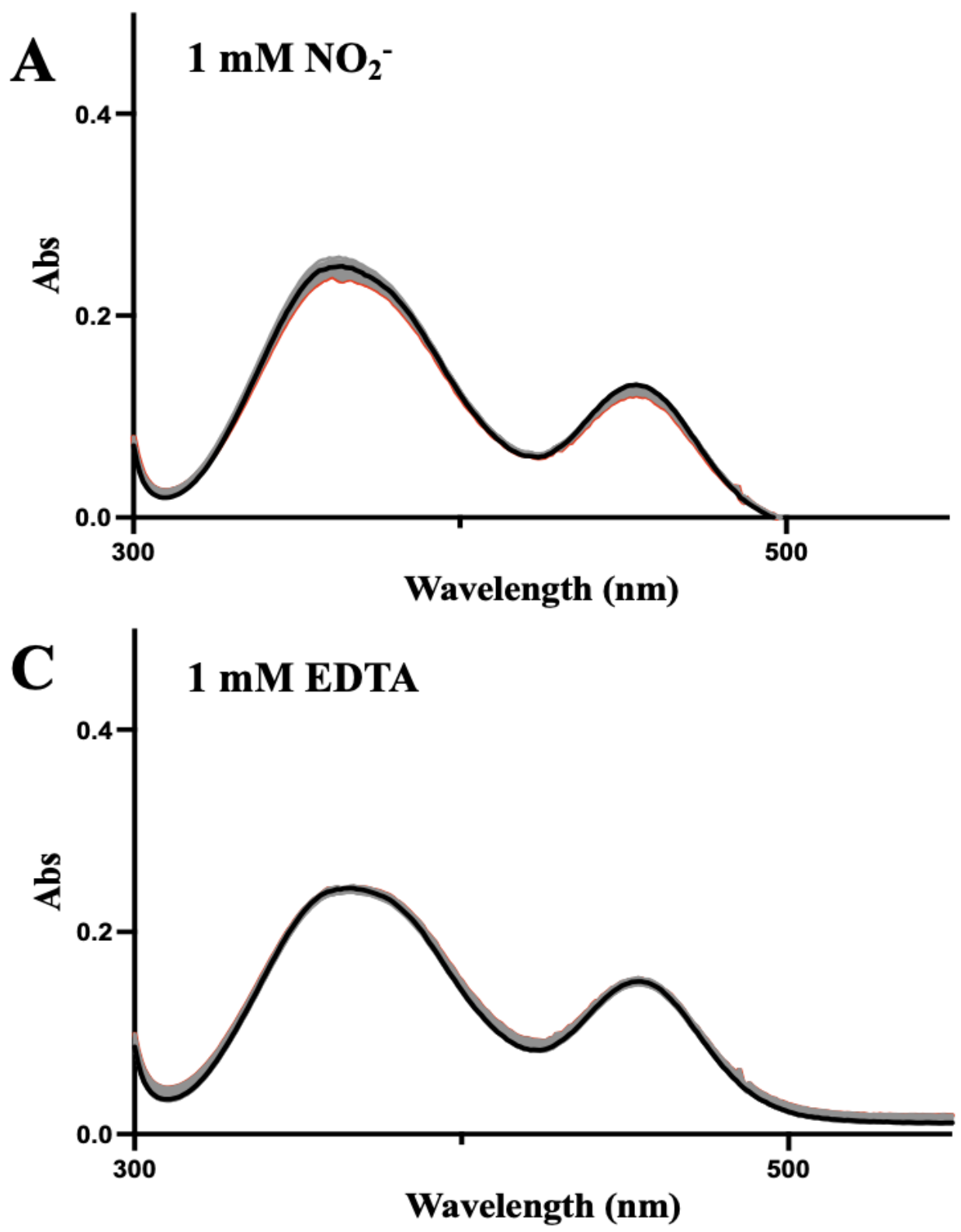

Figure S4. Investigation the effect of sodium nitrite (1 mM, A), sodium nitrate (1 mM, B), EDTA (1 mM, C) and reduced glutathione (2 mM, D) with Ni-Rd (25 μM) in 50 mM sodium phosphate buffer pH 7.0 with 50 mM NaCl; electronic spectra were collected for 1 h at 37 ^{o}C.

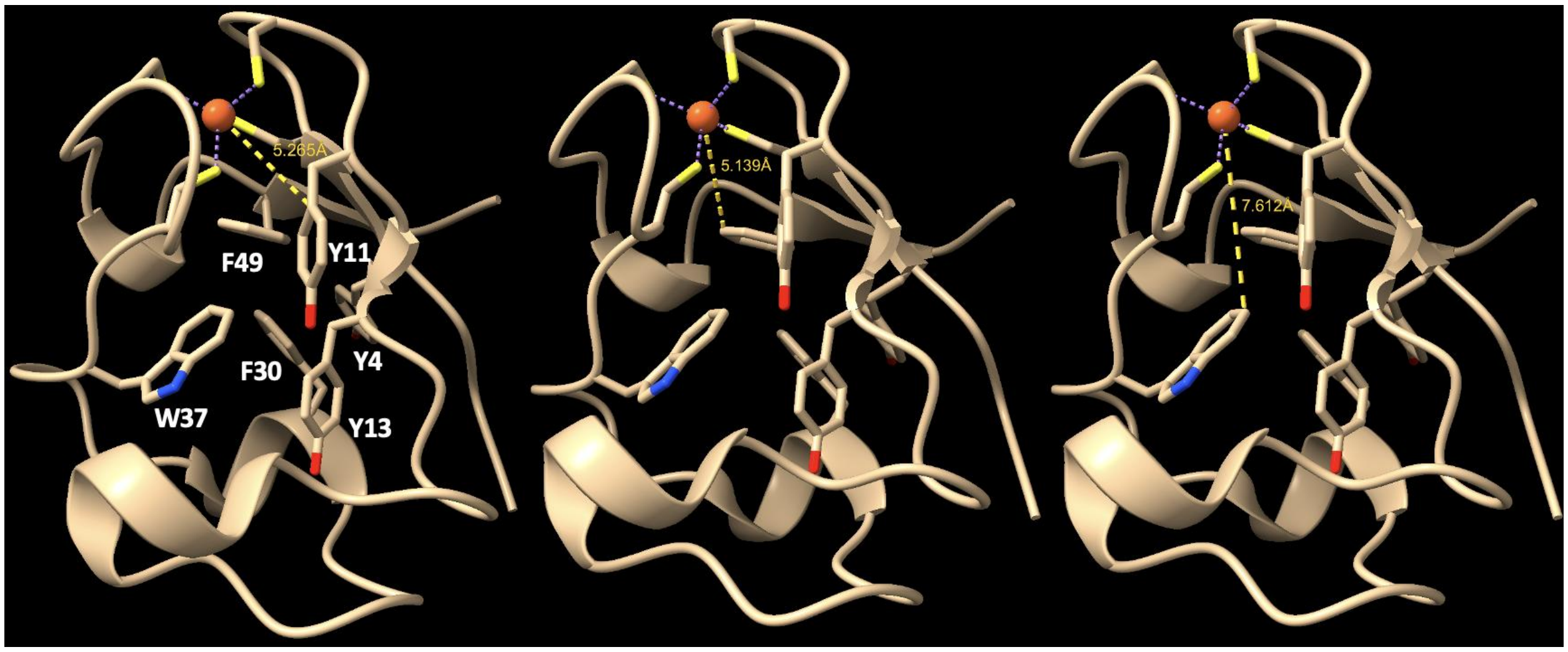

Figure S5. Structure of *D. gigas* rubredoxin (pdb 2DSX) showing the distorted tetrahedral iron site (iron in orange) and all fluorescent residues (tryptophan, tyrosines and phenylalanines) and some of their relative distances to the iron(III).

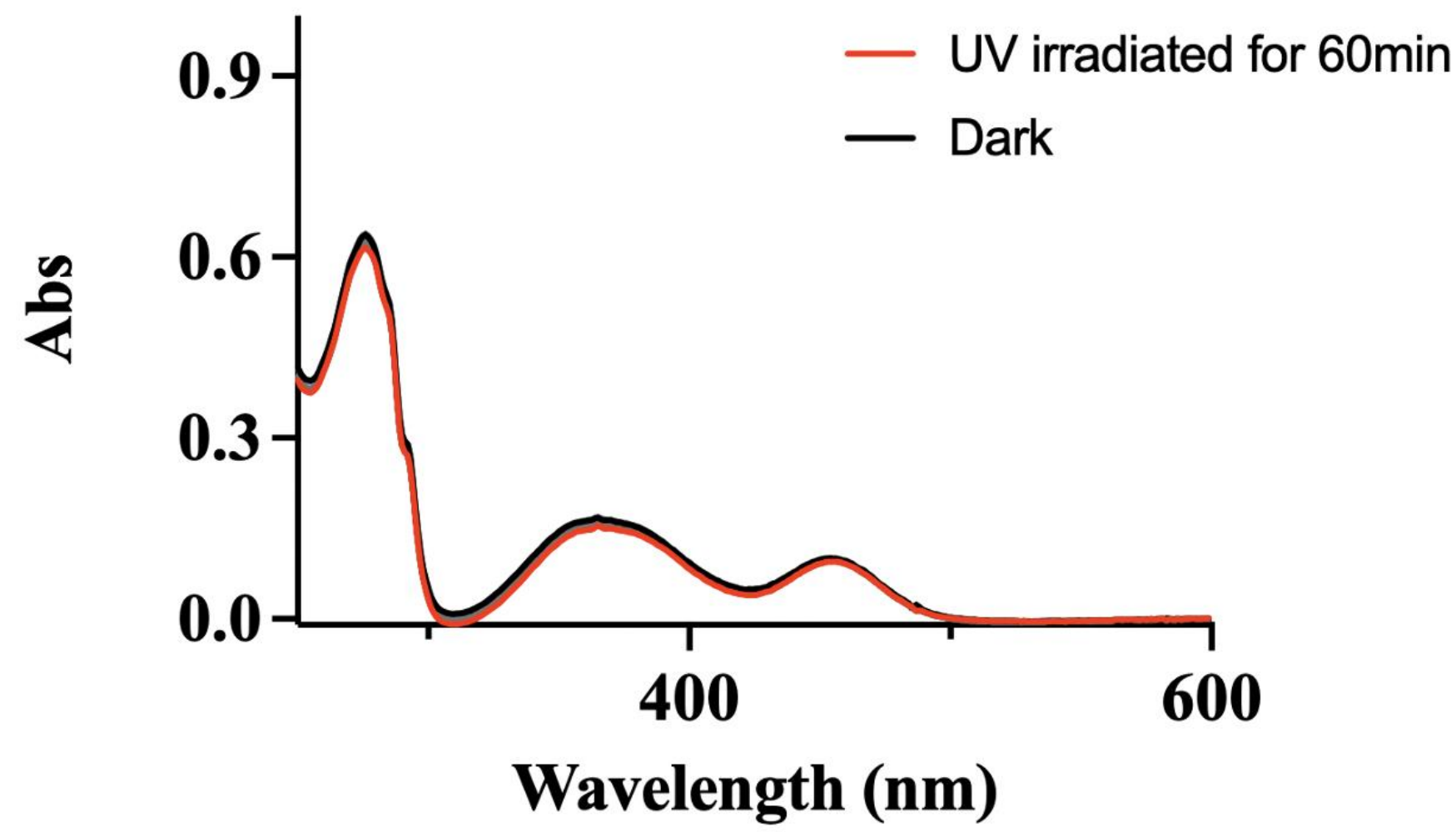

Figure S6. Monitoring the effect of ultraviolet light irradiation on Ni-Rd (25 μM) by electronic spectroscopy, in 50 mM sodium phosphate buffer pH 7.0 with 50 mM NaCl, every 5 min for 1 h.

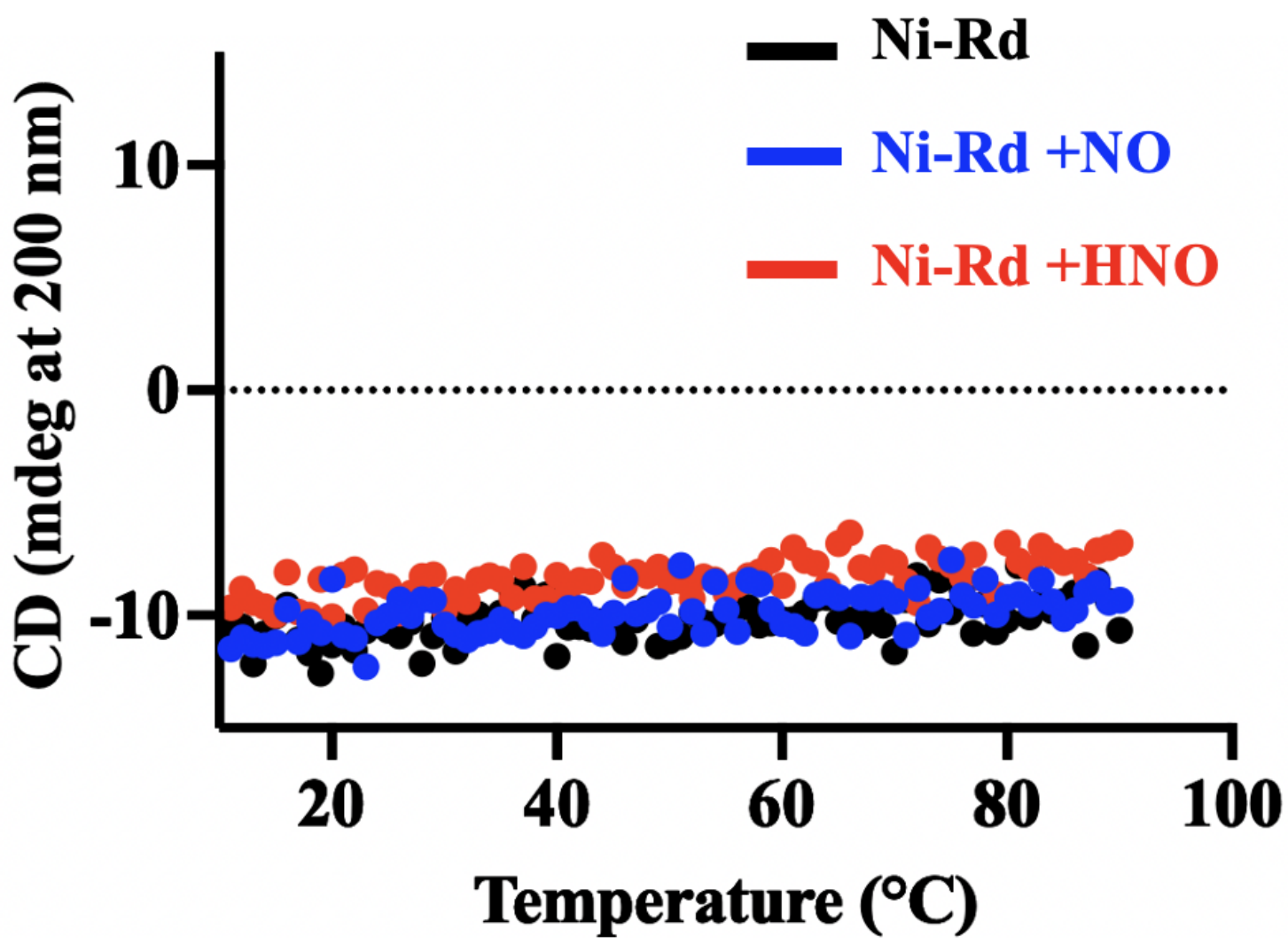

Figure S7. CD investigation of melting denaturation of Ni-Rd with and without NO or HNO in a 5 mM phosphate buffer, pH 7.0. Samples treated with NO or HNO donors (2-fold molar excess) were incubated for 1 h at 37°C before running a thermal study monitoring at 200 nm.

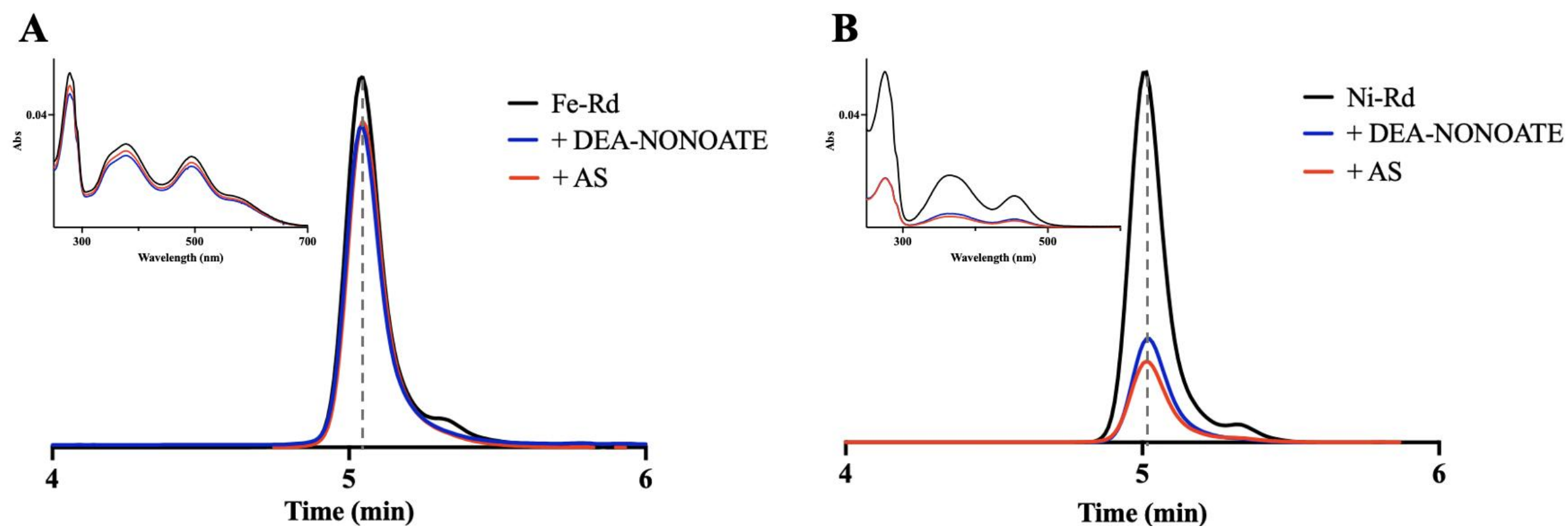

Figure S8. Size-exclusion chromatography study of Fe-Rd (A) and Ni-Rd (B) along with NO and HNO donors

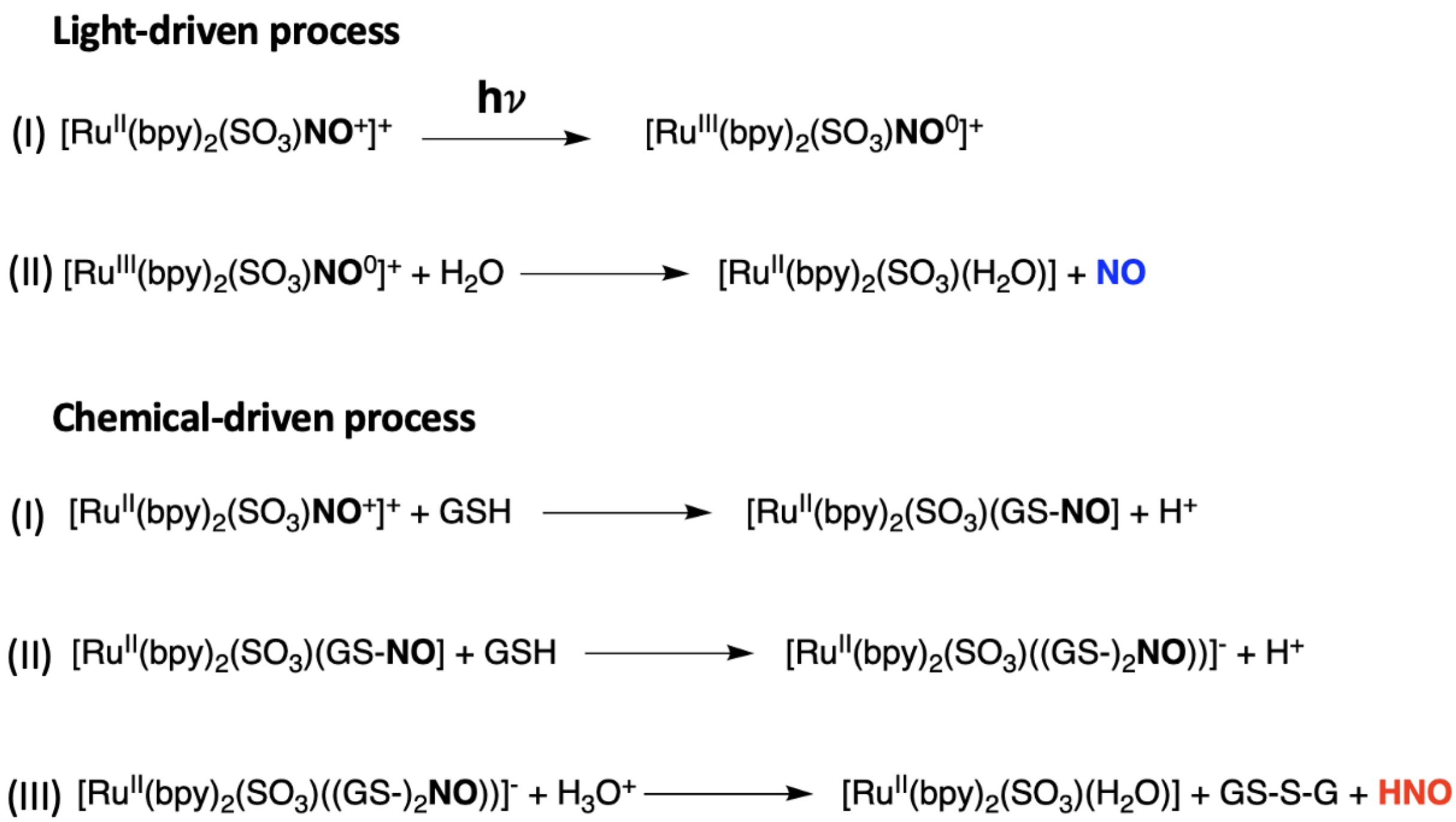

Scheme 1. Mechanism for the production of NO and HNO using $[Ru(bpy)_2(SO_3)NO]^+$ as previously reported in the literature.[1]

Note 2: Some additional experimental details of ICP-AES measurements

Nickel standard solutions were prepared by serial dilution of a certified multi-element standard solution (Reagecon). Table below summarizes the concentrations of the nickel standards used for calibration together with the corresponding AES-ICP signal intensities.

Standard concentration (mg/L)	Intensity (a.u.)
0 (blank)	29,704
0.3125	380,254
0.625	899,834
1.000	1,508,357
1.250	1,896,621
2.000	3,044,402
2.500	3,817,754
4.000	6,378,095
5.000	7,519,637

The calibration plot of Ni^{2+} concentration versus ICP-AES signal intensity for the nickel standard solutions is shown below. This calibration curve was employed to determine the nickel concentration in the protein samples. Linear regression analysis yielded the equation $y = 1{,}550{,}790 \cdot x - 83{,}935$ ($R^2 = 0.998$) over the concentration range investigated.

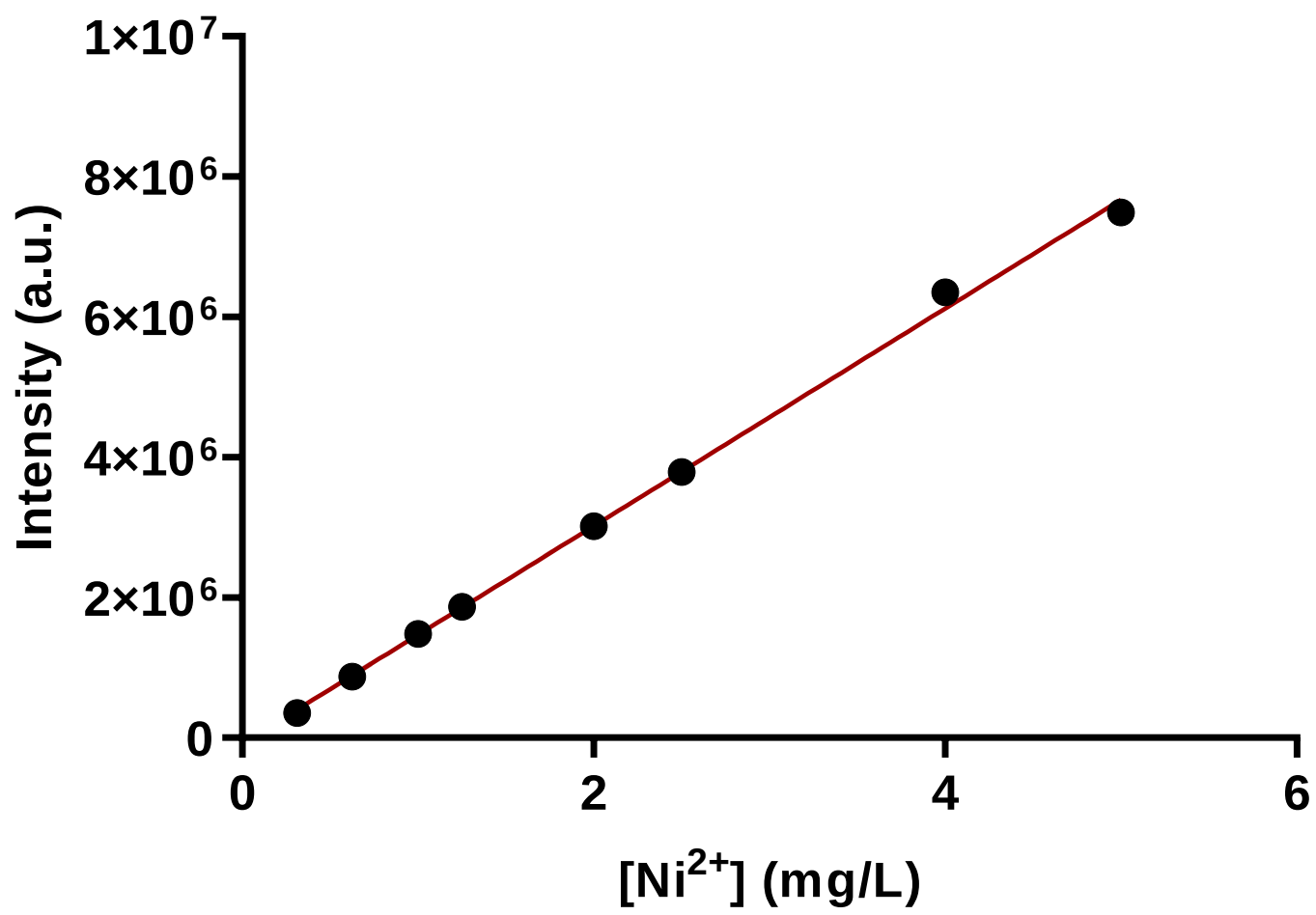

Reference

[1] aE. H. Silva Sousa, L. A. Ridnour, F. S. Gouveia, Jr., C. D. Silva da Silva, D. A. Wink, L. G. de Franca Lopes, P. J. Sadler, *ACS Chem Biol* **2016**, *11*, 2057-2065; bF. O. N. Silva, S. X. B. Araújo, A. K. M. Holanda, E. Meyer, F. A. M. Sales, H. C. N. Diógenes, I. M. M. Carvalho, I. S. Moreira, L. G. F. Lopes, *Eur J Inorg Chem* **2006**, 2020-2026; cM. C. L. Cândido, A. M. Oliveira, F. O. N. Silva, A. K. M. Holanda, W. G. Pereira, E. H. S. Sousa, Z. A. Carneiro, R. S. Silva, L. G. F. Lopes, *J Brazil Chem Soc* **2015**, *26*, 1824-1830; dE. M. Carvalho, E. H. S. Sousa, V. Bernardes-Génisson, L. G. D. Lopes, *Eur J Inorg Chem* **2021**, *2021*, 4316-4348.
